# Current Status of Field-Effect Transistors for Biosensing Applications

**DOI:** 10.3390/bios12121081

**Published:** 2022-11-25

**Authors:** Cristian Ravariu

**Affiliations:** 1 BioNEC Group, Department of Electronic Devices Circuits and Architectures, Polytechnic University of Bucharest, Splaiul Independentei 313, 060042 Bucharest, Romania; cristian.ravariu@upb.ro; 2 EduSciArt SRL, SME of Education Science and Art, Iovita 2, 050686 Bucharest, Romania

The latest novelties in electronic biosensors indicate an increased interest in the compatibilization between Field Effect Transistors (FETs) and bioreceptors, either enzymes, antibodies or cells, for the purpose of detecting the multiple analytes [1,2,3]. Despite existing products based on substrate detection using Enzyme-FETs [4] or antigen detection using Immuno-FETs [5], the spatial coupling of various biodetection materials and nano-scale FETs is a serious challenge. Sometimes, enzymatic receptors need further functionalization, combinations of nano-particles and organic compounds [6,7,8], and other times, they need nano-porous materials anchored in the gate space of an FET transistor [9]. On the other hand, the FET class has been expanded in a huge palette of nano-devices in the last few years [10,11,12]. In biosensing, different transistors have been successfully used, Organic FETs, Carbon Nanotube FETs, Graphene FETs and Silicon-On-Insulator FETs, being the common transistors.

Some nano-transistors have an advantage: they are able to establish an extremely low limit of detection (LOD). This includes Silicon Nanowire FETs [13] or Single Electron Transistors, thanks to their extremely low operation currents [14].

In this Special Issues, recent results of Biosensors based on Field Effect Transistors were reported. The actual topic concerns bio-compatibility issues in enzyme–silicon wafers, biodetection performance, technological aspects, FET biosensors applied in medicine, living matter studies, the food industry, toxicology and environmental analyses, as a few examples.

In this Special Issue, Almeida et al. [15] reported a graphene-based biosensor sensitive to recombinant cyanovirin-N (rCV-N), which is a protein with an antiviral property. The application of this biosensor in medicine is based on its effective microbicide effect to inhibit the replication of human immunodeficiency virus (HIV). The fabrication technology of this graphene-based biosensor is based on a modified graphene monolayer with 1-pyrenebutanoic acid succinimidyl ester that is able to interact with both graphene and the primary and secondary amines of antibodies. The working principle of this biosensor concerns the modification of the electrical resistance of the modified graphene layer. Extremely low detection limits for rCV-N protein in solutions were achieved by this method. Working in the 0.02–10 ng/mL range, the authors found a detection limit of 0.45 pg/mL [15], which is smaller than that obtained with well-known techniques [16]. This result propels the graphene-based biosensors above Silicon Nanowire FETs in terms of performance [17]. From another point of view, a graphene biosensor was studied by Purwidyantri et al. [18] in this Special Issue. In a previous work, the same group of authors reported a graphene-based biosensor that was achieved by CVD grown from a single layer of graphene. It detected DNA molecules up to attomolar concentrations, which represent a signal sensitivity for a single strand of the DNA sequence, using a field effect transistor [19]. A liquid-gated Graphene Field-Effect Transistor (GFET) has been developed in this Special Issue. This set-up can be used with high accuracy for ultrasensitive bio-detection platforms. The characteristics of the GFETs were measured in different conditions, such as different ionic strengths, different pH and electrolyte types, and different buffers, i.e., phosphate buffer (PB) or phosphate buffer saline (PBS). The study shows that a longer Debye length is not the decisive parameter for increasing the sensitivity of the GFET or decreasing its limit of detection. For DNA detection, a prevailing role is fulfilled by the additional salts present in PBS as compared to PB. In this way, the studies have demonstrated a strong increase in the electron mobility in the graphene-based devices [18]. Another topic of this Special Issue concerns the fabrication and modeling of the Enzyme FETs.

A development direction is the down-scaling of the transducer for these kinds of biosensors [20,21], which results in more advantages: co-integration with the Si technology, low limits of detection, reduced costs, integration facilities, low power consumption and increased sensitivity [22]. Some researchers reported the fabrication of Ion-Sensitive Field Effect Transistors (ISFET) able to consume only some pico-watts of energy [23]. The solution for the next ten years seems to be the co-integration of the enzyme receptor with Metal Oxide Semiconductor (MOS) transistors [9] or Organic Thin Film Transistors (OTFT) [24] or any standard Field Effect Transistors (FET) as transducers to produce Enzyme-Field Effect Transistors (ENFET) [25].

In this Special Issue, Ravariu’s group reported a glucose biosensor [26] that was optimized in the second iteration, starting from a previous experience [9]. A standard MOS-FET transistor was co-integrated with an enzymatic membrane made by glucose oxidase enzyme entrapped in nafion. To optimize the enzyme immobilization, a titanium film was deposited over the gate metal, and then it was converted by anodization into TiO_2_nanotubes. The paper described multiple details of the co-integration technology between MOS devices and enzymatic receptors. Finally, the ENFET was tested at V_DS_ = 2 V and V_GS_ = 4 V. The optimal saturation regime for a good linearity of the biosensor seems to be in the glucose concentration range of 0.001 mM–100 mM, which is larger than the linear range of other glucose biosensors [27].

Models of the enzymatic kinetic within an ENFET were developed in a second paper [28]. Software tools able to simulate the functionality of ENFETs are missing. For instance, the Silvaco platform has resources to simulate optical sensors, magneto-transistors and very recent electrochemical sensors [29], but it does not have facilities to simulate biosensors.

The international community has been reporting the lack of models for Bio-FETs for many years, promoting any advancement [30]. The last paper from this issue generalizes previous concepts, extending the reaction rate from first-order to second-order and to n-order, where n > 1. The developed model envisaged three stages: (1) the dependence of the ion product’s concentration on the initial glucose concentration, as a consequence of the enzymatic reaction; (2) the shift in the threshold voltage versus the accumulated ions in the gate space; and (3) the drain current deviation under the control of the gate voltage.

This Special Issue included important contributions on transistors for biosensing applications, including graphene-based biosensors, liquid-gated graphene field-effect transistors and technological solutions for MOS-FET co-integration and Enzyme FET modeling. The results are promising. They prove a detection limit up to attomolar concentrations, while enzyme immobilization techniques appeal to both cross-linked and nano-structured materials.
